# A positive feedback loop between ZNF205‐AS1 and EGR4 promotes non‐small cell lung cancer growth

**DOI:** 10.1111/jcmm.14056

**Published:** 2018-12-16

**Authors:** Susu He, Jian Lin, Youzu Xu, Ling Lin, Jiaxi Feng

**Affiliations:** ^1^ Department of Respiratory Medicine Taizhou Hospital of Wenzhou Medical University Linhai Zhejiang China

**Keywords:** EGR4, feedback loop, growth, long noncoding RNA, non‐small cell lung cancer

## Abstract

Accumulating evidences revealed that long noncoding RNAs (lncRNAs) are frequently implicated in non‐small cell lung cancer (NSCLC). Herein, we reported the identification of a novel NSCLC‐associated functional lncRNA ZNF205 antisense RNA 1 (ZNF205‐AS1). ZNF205‐AS1 was increased in NSCLC tissues and cell lines, and associated with poor prognosis of NSCLC patients. Bioinformatics prediction, combined with experimental verification revealed that early growth response 4 (EGR4) directly bound to *ZNF205‐AS1* promoter, increased the promoter activity of *ZNF205‐AS1*, and activated *ZNF205‐AS1* transcription. Intriguingly, ZNF205‐AS1 transcript directly interacted with EGR4 mRNA, increased EGR4 mRNA stability, and up‐regulated EGR4 expression via RNA‐RNA interaction. Thus, ZNF205‐AS1 and EGR4 formed a positive feedback loop. Through regulating EGR4, ZNF205‐AS1 activated its own promoter activity. EGR4 was also increased in NSCLC and the expression of ZNF205‐AS1 was significantly positively correlated with EGR4 in NSCLC tissues. Gain‐of‐function and loss‐of‐function assays demonstrated that both ZNF205‐AS1 and EGR4 promoted NSCLC cell growth in vitro and NSCLC tumour growth in vivo. Concurrently depleting ZNF205‐AS1 and EGR4 more significantly repressed NSCLC tumour growth in vivo. Collectively, our study demonstrated that the positive feedback loop between ZNF205‐AS1 and EGR4 promotes NSCLC growth, and implied that targeting this feedback loop may be promising therapeutic strategy for NSCLC.

## 1 INTRODUCTION

Lung cancer is the most common diagnosed cancer worldwide, which accounts for 11.6% of total cancer cases.^1^ Lung cancer is also the leading cause of cancer‐related death worldwide, which accounts for 18.4% of total cancer deaths.^1^ Non‐small cell lung cancer (NSCLC) is the major type of lung cancer, which accounts for about 85% of all lung cancer cases.^2^ Despite great advances in therapies for NSCLC, including surgical resection, chemotherapy, radiotherapy, immunotherapy, and molecular targeted therapy, the prognoses of NSCLC patients are still dismal with a 5‐year survival rate of about 15%.^3^ Therefore, further revealing the molecular mechanisms underlying the initiation and progression of NSCLC and developing more effective therapies are necessary.

Former investigations have identified several genomic mutations, such as *EGFR*,* P53*, and *KRAS*.^4^ Recently, high throughput transcriptome sequencings have identified more aberrations in gene expression.^5^ Among these aberrantly expressed transcripts, long noncoding RNAs (lncRNAs) have attracted increasing attentions.^6^ LncRNA is a class of transcript with more than 200 nucleotides in length, but do not encode proteins.^7^, ^8^, ^9^ Aberrant expressions of lncRNAs have been revealed in various pathological status, particular in cancers.^10^, ^11^, ^12^, ^13^ Furthermore, lncRNAs also play important roles in various pathophysiological processes, including cancers.^14^, ^15^, ^16^, ^17^, ^18^ In NSCLC, several lncRNAs are reported to participate in tumourigenesis and/or development of NSCLC. LncRNA MALAT‐1 is frequently revelated to regulate NSCLC metastasis.^19^ LncRNA MEG3 is reported to regulate cisplatin resistance of NSCLC.^20^ MetaLnc9 promotes NSCLC metastasis via activation of AKT/mTOR pathway.^21^ LncRNA VELUCT regulates NSCLC cell viability.^22^ Knockdown of LINC01614 inhibits NSCLC progression.^23^

Compared with these limited number of lncRNAs reported to play roles in NSCLC, more lncRNAs are revealed to be aberrantly expressed in NSCLC.^24^ Among these thousands of lncRNAs aberrantly expressed in NSCLC, many lncRNAs may also have critical roles, which need further investigation. Seiler et al performed a functional siRNA screen to search the lncRNAs regulating NSCLC cell viability.^22^ Among the list of candidate targets, we noted ZNF205 antisense RNA 1 (ZNF205‐AS1) (NCBI Reference Sequence: NR_024167.1), which has a relative high score in the screen, but whose roles in cancers are unknown.

In this study, we further investigated the expression pattern and biological roles of ZNF205‐AS1 in NSCLC. We also explored the reason contributing to the aberrant expression of ZNF205‐AS1 in NSCLC. Intriguingly, we identified a positive feedback loop between ZNF205‐AS1 and transcription factor Early Growth Response 4 (EGR4) in NSCLC, which significantly promoted NSCLC growth.

## 2 MATERIALS AND METHODS

### 2.1 Clinical samples

A total of 90 pairs of NSCLC tissues and matched adjacent noncancerous lung tissues were obtained from NSCLC patients, who received surgical resection at Taizhou Hospital of Wenzhou Medical University (Linhai, China). All tissues were diagnosed by pathological examination and preserved at −80°C for subsequent analysis. The Ethics Committee of Taizhou Hospital of Wenzhou Medical University (Linhai, China) reviewed and approved this study. Written informed consent was acquired from all patients.

### 2.2 Cell cultures and treatments

Human normal bronchial epithelial cell line 16HBE and NSCLC cell lines PC‐9, NCI‐H1299, NCI‐H23, SK‐MES‐1, and SPC‐A1 were obtained from the Institute of Biochemistry and Cell Biology of the Chinese Academy of Science (Shanghai, China). 16HBE and PC‐9 cells were cultured in Dulbecco's Modified Eagle's Medium (DMEM) (Invitrogen, Carlsbad, CA, USA). NCI‐H1299, NCI‐H23, and SPC‐A1 cells were cultured in RPMI‐1640 Medium (Invitrogen). SK‐MES‐1 cells were cultured in Eagle's Minimum Essential Medium (MEM) (Invitrogen). All the cells were grown in the above described medium supplemented with 10% foetal bovine serum (FBS) (Invitrogen) at 37°C with 5% CO_2_. Where indicated, NSCLC cells were treated with 50 µM α‐amanitin (Sigma‐Aldrich, Saint Louis, MO, USA) for 0‐24 hours.

### 2.3 Vectors construction and transfection

The complementary DNA (cDNA) encoding ZNF205‐AS1 was PCR‐amplified using the Thermo Scientific Phusion Flash High‐Fidelity PCR Master Mix (Thermo‐Fisher Scientific, Waltham, MA, USA) and the primers 5'‐CCCAAGCTTGAAAGCGCCTCTTCCCCGT‐3' (forward) and 5'‐CGGGATCCCCCAGGAGTAATTTTTCTTTCAT‐3' (reverse). The PCR products were cloned into the Hind III and BamH I sites of pcDNA3.1(+) (Invitrogen) or pSPT19 (Roche, Mannheim, Germany) vectors, termed as pcDNA3.1‐ZNF205‐AS1 or pSPT19‐ZNF205‐AS1, respectively. The cDNA encoding EGR4 was PCR‐amplified using the Thermo Scientific Phusion Flash High‐Fidelity PCR Master Mix and the primers 5'‐CGGGATCCTGTTTGGGCATTTACGTCAC‐3' (forward) and 5'‐GGAATTCGGAGGAGTTGGAAGAAGAGCG‐3' (reverse). The PCR products were cloned into the BamH I and EcoR I sites of pcDNA3.1(+) (Invitrogen) vector, termed as pcDNA3.1‐EGR4.

The cDNA oligonucleotides suppressing ZNF205‐AS1 expression were synthesized by GenePharma (Shanghai, China) and inserted into the GenePharma SuperSilencing^TM^ shRNA expression vector pGPH1/Neo. The shRNAs target sites for ZNF205‐AS1 were 5'‐GCUUGAAUAGUGUCCUCUAAG‐3' (sh‐ZNF205‐AS1‐1) and 5'‐GGAGUCCUGGGAUUCUGAUUG‐3' (sh‐ZNF205‐AS1‐2). The cDNA oligonucleotides suppressing EGR4 expression were synthesized by GenePharma and inserted into the GenePharma SuperSilencing^TM^ shRNA expression vector pGPH1/Hygro. The shRNAs target sites for EGR4 were 5'‐GGACCAAGAUUGAGGACUU‐3' (sh‐EGR4‐1) and 5'‐GCUACAGCGGUAGCUUCUU‐3' (sh‐EGR4‐2).^25^

The promoter region of *ZNF205‐AS1*, from −1025 to +47 base pair (bp) upstream of the transcription start site, was PCR‐amplified using the Thermo Scientific Phusion Flash High‐Fidelity PCR Master Mix and the primers 5'‐CTAGCTAGCGGAAAGAGGAGACGGCAGAGCA‐3' (forward) and 5'‐CCCAAGCTTTGGCGGAGGTAGGAGAGGGA‐3' (reverse). The PCR products were cloned into the Nhe I and Hind III sites of pGL3‐Basic Vector (Promega, Madison, WI, USA), termed as pGL3‐ZNF205‐AS1.

The transfection and cotransfection of vectors were performed using Lipofectamine 3000 (Invitrogen) according to the protocol.

### 2.4 Stable cell lines construction

To obtain ZNF205‐AS1 or EGR4 stably overexpressed cells, pcDNA3.1‐ZNF205‐AS1 or pcDNA3.1‐EGR4 was transfected into PC‐9 cells. 48 hours after transfection, the cells were selected with neomycin for 4 weeks. To obtain ZNF205‐AS1 stably depleted cells, sh‐ZNF205‐AS1‐1 or sh‐ZNF205‐AS1‐2 was transfected into SPC‐A1 cells. 48 hours after transfection, the cells were selected with neomycin for 4 weeks. To obtain EGR4 stably depleted cells, sh‐EGR4‐1 or sh‐EGR4‐2 was transfected into SPC‐A1 cells. 48 hours after transfection, the cells were selected with hygromycin for 4 weeks. To obtain EGR4 and ZNF205‐AS1 concurrently stably depleted cells, sh‐EGR4‐2 and sh‐ZNF205‐AS1‐1 were cotransfected into SPC‐A1 cells. 48 hours after transfection, the cells were selected with neomycin and hygromycin for 4 weeks.

### 2.5 RNA isolation, reverse transcription, and quantitative polymerase chain reaction (qPCR)

Total RNA was isolated from indicated tissues or cells with the TRIzol Reagent (Invitrogen) according to the protocol. After being treated with DNase I (Takara, Dalian, China) to remove genomic DNA, the purified RNA was used to perform reverse transcription with the M‐MLV Reverse Transcriptase (Invitrogen) to generate first‐stand cDNA. The first‐stand cDNA was used to perform quantitative polymerase chain reaction (qPCR) with SYBR^®^ Premix Ex Taq™ II kit (Takara) on StepOnePlus Real‐Time PCR System (Applied Biosystems, Foster City, CA, USA) according to the standard SYBR Green protocol. The sequences of primers used for qPCR were: for ZNF205‐AS1, 5'‐AGAATGGGACCTTATTGGG‐3' (forward) and 5'‐ATGGGAAGAAGAGCGAAAC‐3' (reverse); for EGR4, 5'‐AGCAAGAGATGGGTTTATG‐3' (forward) and 5'‐AGGAGTTGGAAGAAGAGC‐3' (reverse); for U6, 5'‐GCTTCGGCAGCACATATACTAAAAT‐3' (forward) and 5'‐CGCTTCACGAATTTGCGTGTCAT‐3' (reverse); for 18S rRNA, 5'‐ACACGGACAGGATTGACAGA‐3' (forward) and 5'‐GGACATCTAAGGGCATCACA‐3' (reverse); for β‐actin, 5'‐GGGAAATCGTGCGTGACATTAAG‐3' (forward) and 5'‐TGTGTTGGCGTACAGGTCTTTG‐3' (reverse). β‐actin was used as an endogenous control for the quantification of RNAs expression. The quantification of RNAs expression was calculated according to the comparative Ct method.

### 2.6 Western blot

Total cell lysates were extracted from indicated NSCLC cells using RIPA lysis buffer (Beyotime, Shanghai, China) supplemented with protease inhibitors (Beyotime) following the protocol. Identical quantities of protein samples were separated by 12% sodium dodecyl sulphate‐polyacrylamide gel electrophoresis (SDS‐PAGE) and transferred onto nitrocellulose membrane (Millipore, Bedford, MA, USA). After being blocked using 5% nonfat milk, the membranes were incubated with primary antibodies against EGR4 (Abcam, Hong Kong, China) or β‐actin (Proteintech, Rosemont, IL, USA). After three washes, the membranes were incubated with IRdye 700‐conjugated goat anti‐mouse IgG or IRdye 800‐conjugated goat anti‐rabbit IgG second antibodies and screened on an Odyssey infrared scanner (Li‐Cor, Lincoln, NE, USA).

### 2.7 Chromatin immunoprecipitation (ChIP) assay

ChIP assay was carried out in indicated NSCLC cells with the EZ‐Magna ChIP™ A/G Chromatin Immunoprecipitation Kit (Millipore) and EGR4 antibody (Abcam) according to the instructions. The retrieved DNA was detected by qPCR as above described to measure the enrichment of *ZNF205‐AS1* promoter. The sequences of primers used were: F1, 5'‐AGGGCTGTGGGAGGAGAGA‐3'; R1, 5'‐GTGGGGAGGTGGAGGTTTG‐3'; F2, 5'‐ATCACGCCACTACACTCCA‐3'; R2, 5'‐TGACTCCTCAATTCCAGACT‐3'.

### 2.8 Dual luciferase reporter assay

pcDNA3.1‐ZNF205‐AS1, pcDNA3.1‐EGR4, or pcDNA3.1 was cotransfected with pGL3‐ZNF205‐AS1 or pGL3‐Basic and pRL‐TK vector which expresses renilla luciferase into PC‐9 cells. sh‐ZNF205‐AS1‐1, sh‐ZNF205‐AS1‐2, sh‐EGR4‐1, sh‐EGR4‐2, or sh‐NC was cotransfected with pGL3‐ZNF205‐AS1 or pGL3‐Basic and pRL‐TK vector into SPC‐A1 cells. 48 hours after transfection, the firefly luciferase and renilla luciferase activity were measured using the Dual‐Luciferase^®^ Reporter Assay System (Promega) according to the protocol. Renilla luciferase activity was used as an endogenous control for the quantification of firefly luciferase activity.

### 2.9 Purification of nuclear and cytoplasmic RNA

Nuclear and cytoplasmic RNA were purified with the Cytoplasmic & Nuclear RNA Purification Kit (Norgen, Belmont, CA, USA) according to the instruction. Briefly, SPC‐A1 cells were lysed and centrifuged. Cytoplasmic RNA exists in the supernatant, and while nuclear RNA exists in the pellet. The RNA in both fractions was bound and purified with the columns provided in this kit.

### 2.10 RNA pull‐down assay

ZNF205‐AS1 was in vitro transcribed from pSPT19‐ZNF205‐AS1 and biotin‐labelled using the Biotin RNA Labelling Mix (Roche) and T7 RNA polymerase (Roche) according to the protocols. ZNF205‐AS1 antisense RNA was in vitro transcribed from pSPT19‐ZNF205‐AS1 and biotin‐labelled using the Biotin RNA Labeling Mix (Roche) and SP6 RNA polymerase (Roche) according to the protocols. After being treated with DNase I (Takara), the in vitro transcribed RNAs were purified using the RNeasy Mini Kit (Qiagen, Valencia, CA, USA) according to the protocols. Then, 3 µg of purified biotin‐labelled RNAs were incubated with 1 mg of SPC‐A1 whole‐cell lysates at 25°C for 1 hour. Next, the complexes were isolated using streptavidin agarose beads (Invitrogen). The RNAs enriched in the pulldown material were detected by qPCR as above described.

### 2.11 Cell growth assay

Glo cell viability assay and Ethynyl deoxyuridine (EdU) immunofluorescence staining were undertaken to evaluate NSCLC cell growth. For Glo cell viability assay, 3000 indicated NSCLC cells/well were plated into 96‐well plates. At indicated time after plating, cell viabilities were evaluated with the CellTiter‐Glo Luminescent Cell Viability Assay (Promega) following the instruction. EdU immunofluorescence staining was undertaken with the EdU kit (RiboBio, Guangzhou, China) following the instruction. The results were collected using the Zeiss Photomicroscope (Carl Zeiss, Oberkochen, Germany) and quantified via counting at least ten random fields.

### 2.12 Xenograft assay

Five‐week‐old male BALB/c‐nu/nu nude mice were purchased from SLRC Laboratory Animal Center (Shanghai, China) and grown in the pathogen‐free condition for xenograft assays. The Ethics Committee of Taizhou Hospital of Wenzhou Medical University (Linhai, China) reviewed and approved the use of animals. 3×10^6^ indicated NSCLC cells were subcutaneously injected into the flanks of these mice. The growth of subcutaneous tumours was detected every three days using a caliper, and calculated following the equation V = a×b^2^/2 (a, long axes; b, short axes).

### 2.13 Ki67 Immunohistochemistry (IHC) and TUNEL staining

Ki67 immunohistochemistry (IHC) was undertaken on paraffin embedded sections of subcutaneous xenografts and clinical NSCLC tissues with Ki67 primary antibody (Abcam) and a horseradish peroxidase‐conjugated secondary antibody (Invitrogen). The proteins in situ were visualized with 3, 3‐diaminobenzidine. Terminal deoxynucleotidyl transferase (TdT)‐mediated dUTP nick end labelling (TUNEL) staining was undertaken on paraffin embedded sections of subcutaneous xenografts using the In‐Situ Cell Death Detection Kit (Roche) according to the protocol.

### 2.14 Senescence‐associated β‐galactosidase (SA‐β‐gal) staining

Cellular senescence of indicated NSCLC cells was evaluated using Senescence‐associated β‐galactosidase (SA‐β‐gal) staining with the Senescence β‐Galactosidase Staining Kit (Beyotime) in accordance with the protocol. The results were collected using the Zeiss Photomicroscope and quantified via counting at least 10 random fields.

### 2.15 Statistical analysis

Statistical analyses were undertaken with the SPSS 18.0 software package (Chicago, IL, USA). For comparisons, Wilcoxon signed‐rank test, Pearson chi‐square test, Log‐rank test, one‐way ANOVA followed by Dunnett's multiple comparison test, Student's *t* test, Pearson correlation analysis, and Mann‐Whitney test were undertaken as indicated. *P *< 0.05 was considered as statistically significant.

## 3 RESULTS

### 3.1 ZNF205‐AS1 was increased in NSCLC and correlated with poor prognosis of NSCLC patients

**Figure 1:**
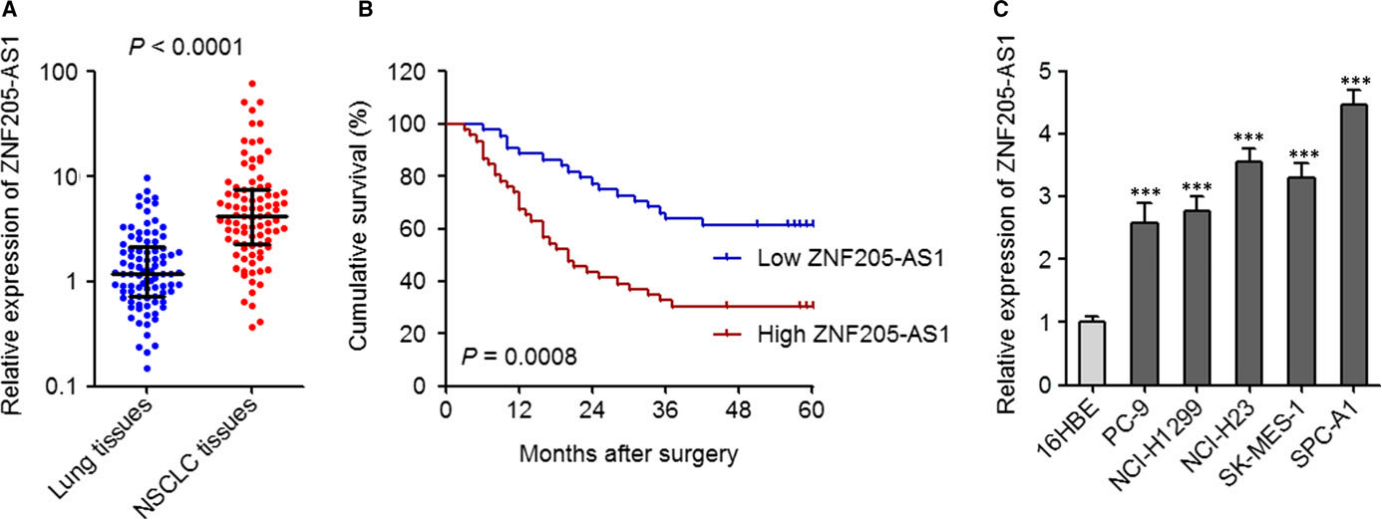
ZNF205‐AS1 was increased in NSCLC and correlated with poor prognosis of NSCLC patients. A, The expression of ZNF205‐AS1 in 90 pairs of NSCLC tissues and matched adjacent noncancerous lung tissues was quantified by qPCR. Results are displayed as median with interquartile range. *P* < 0.0001 by Wilcoxon signed‐rank test. B, Kaplan‐Meier survival analyses of the correlations between ZNF205‐AS1 expression level and overall survival of these 90 NSCLC patients. ZNF205‐AS1 median expression level was used as cut‐off. *P* = 0.0008 by log‐rank test. C, The expression of ZNF205‐AS1 in normal bronchial epithelial cell line 16HBE and NSCLC cell lines PC‐9, NCI‐H1299, NCI‐H23, SK‐MES‐1, and SPC‐A1 was quantified by qPCR. Results are displayed as mean ± SD of three independent experiments. ***P < 0.001 by one‐way ANOVA followed by Dunnett's multiple comparison test

To investigate the expression pattern of ZNF205‐AS1 in NSCLC, we collected 90 pairs of NSCLC tissues and matched adjacent noncancerous lung tissues, and measured the expression of ZNF205‐AS1 in these tissues using qPCR. As presented in Figure 1A, ZNF205‐AS1 was significantly increased in NSCLC tissues compared with adjacent noncancerous lung tissues. Correlation regression analyses of the association between ZNF205‐AS1 expression levels and clinicopathological characteristics of NSCLC patients displayed that increased ZNF205‐AS1 expression levels were associated with poor pathological differentiation (*P* = 0.035), great tumour diameter (*P* = 0.049), lymph nodes metastasis (*P* = 0.033), and advanced TNM stage (*P* = 0.020) (Table 1). Furthermore, Kaplan‐Meier survival analyses in these 90 NSCLC patients displayed that increased ZNF205‐AS1 expression levels were associated with poor overall survival (*P* = 0.0008) (Figure 1B). In addition, ZNF205‐AS1 expression levels in normal bronchial epithelial cell line 16HBE and NSCLC cell lines PC‐9, NCI‐H1299, NCI‐H23, SK‐MES‐1, and SPC‐A1 was measured using qPCR. As presented in Figure 1C, ZNF205‐AS1 was consistently increased in NSCLC cell lines compared with normal bronchial epithelial cell line. Collectively, these results demonstrated the increased expression of ZNF205‐AS1 in NSCLC and the association between ZNF205‐AS1 and poor prognosis of NSCLC patients.

**Table 1:** Correlation between ZNF205‐AS1 expression levels and clinicopathological characteristics of NSCLC patients

Characteristics	ZNF205‐AS1		*χ* ^2^	*P* value^a^
	Low	High		
Gender			0.185	0.667
Male	26	28		
Female	19	17		
Age (y)			0.407	0.523
>60	24	27		
≤60	21	18		
Histologic subtype			0.179	0.673
Squamous cell carcinoma	23	25		
Adenocarcinoma	22	20		
Pathological differentiation			4.464	0.035*
High‐middle	29	19		
Low	16	26		
Maximum diameter (cm)			3.876	0.049*
≤3	33	24		
>3	12	21		
Lymph nodes metastasis			4.555	0.033*
Negative	31	21		
Positive	14	24		
TNM stage			5.378	0.020*
I	28	17		
II‐III	17	28		

a ZNF205‐AS1 median expression level was used as cut‐off.

*P* value was obtained by Pearson chi‐square test.

^*^ *P < *0.05.

### 3.2 EGR4 directly activated the transcription of *ZNF205‐AS1*

**Figure 2:**
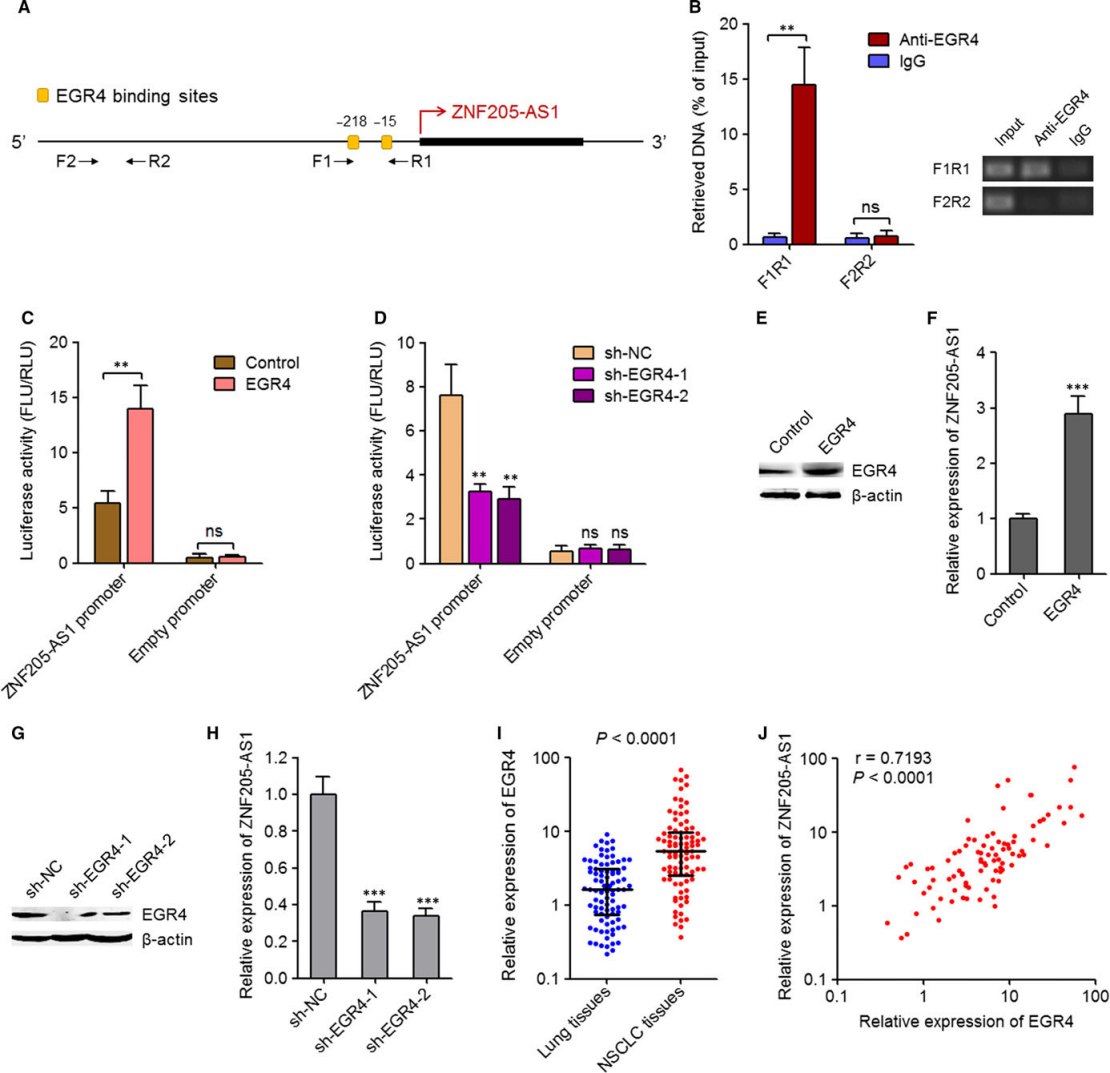
EGR4 activated *ZNF205‐AS1* transcription. A, Schematic diagram of the predicted EGR4 binding sites at the promoter of *ZNF205‐AS1*. B, ChIP assay was carried out using EGR4 specific antibody in SPC‐A1 cells. The retrieved DNA was quantified by qPCR to detect the occupation of EGR4 on the promoter of *ZNF205‐AS1*. Results are displayed as percentage of input DNA and mean ± SD of three independent experiments. **P < 0.01, ns, not significant, by Student's *t* test. C, Luciferase reporter assay in PC‐9 cells cotransfected with the *ZNF205‐AS1* promoter reporter construct and EGR4 overexpression plasmid. The ratios of firefly luciferase (FLU) activity to Renilla luciferase (RLU) activity are displayed. Results are displayed as mean ± SD of three independent experiments. **P < 0.01, ns, not significant, by Student's *t* test. D, Luciferase reporter assay in SPC‐A1 cells cotransfected with the *ZNF205‐AS1* promoter reporter construct and EGR4 specific shRNAs. The ratios of firefly luciferase (FLU) activity to Renilla luciferase (RLU) activity are displayed. Results are displayed as mean ± SD of three independent experiments. **P < 0.01, ns, not significant, by one‐way ANOVA followed by Dunnett's multiple comparison test. E, The expression of EGR4 in EGR4 stably overexpressed and control PC‐9 cells was detected by western blot. F, The expression of ZNF205‐AS1 in EGR4 stably overexpressed and control PC‐9 cells was detected by qPCR. Results are displayed as mean ± SD of three independent experiments. ***P < 0.001 by Student's *t* test. G, The expression of EGR4 in EGR4 stably depleted and control SPC‐A1 cells was detected by western blot. H, The expression of ZNF205‐AS1 in EGR4 stably depleted and control SPC‐A1 cells was detected by qPCR. Results are displayed as mean ± SD of three independent experiments. ***P < 0.001 by one‐way ANOVA followed by Dunnett's multiple comparison test. I, The expression of EGR4 in 90 pairs of NSCLC tissues and matched adjacent noncancerous lung tissues was quantified by qPCR. Results are displayed as median with interquartile range. *P* < 0.0001 by Wilcoxon signed‐rank test. J, The expression levels of EGR4 and ZNF205‐AS1 were significantly positively correlated in these 90 NSCLC tissues. *r* = 0.7193, *P < *0.0001 by Pearson correlation analysis

To investigate the reasons contributing to the elevation of ZNF205‐AS1 in NSCLC, we screened the promoter region of *ZNF205‐AS1* using JASPAR (http://jaspar.genereg.net/),^26^ and predicted two EGR4 binding sites, locating at −15 and −218 upstream of the transcription start site of *ZNF205‐AS1* (Figure 2A). To investigate whether EGR4 binds to the predicted sites at *ZNF205‐AS1* promoter, ChIP assays were carried out with EGR4 specific antibody. As presented in Figure 2B, the *ZNF205‐AS1* promoter region containing the predicted binding sites was specific enriched by EGR4 specific antibody, whereas a distal region of *ZNF205‐AS1* promoter without the EGR4 binding sites was not enriched. To further investigate whether EGR4 modulates the transcriptional activity of *ZNF205‐AS1* via binding to *ZNF205‐AS1* promoter, dual luciferase reporter assays were carried out. The promoter of *ZNF205‐AS1* from −1025 to +47 bp upstream of the transcription start site was cloned into the pGL3‐basic firefly luciferase reporter. The constructed or empty pGL3‐basic firefly luciferase reporter was cotransfected with EGR4 overexpression or empty plasmids into PC‐9 cells. As presented in Figure 2C, ectopic expression of EGR4 increased luciferase activity of the constructed reporter. In addition, the constructed luciferase reporter was cotransfected with two independent EGR4 specific shRNAs into SPC‐A1 cells. The results displayed that knockdown of EGR4 by two independent shRNAs both decreased luciferase activity of the constructed reporter (Figure 2D). These results suggested that EGR4 activated the promoter activity of *ZNF205‐AS1*. Next, the effects of EGR4 on the expression of ZNF205‐AS1 was detected. We constructed EGR4 that stably overexpressed and control PC‐9 cells via transfecting EGR4 overexpression and empty plasmids, respectively. The overexpression efficiency was verified using western blot (Figure 2E). The expression of ZNF205‐AS1 in EGR4 stably overexpressed and control PC‐9 cells was measured using qPCR. As displayed in Figure 2F, ectopic expression of EGR4 up‐regulated ZNF205‐AS1. We also stably depleted EGR4 in SPC‐A1 cells via transfecting two independent ZNF205‐AS1 specific shRNAs. The knockdown efficiency was verified using western blot (Figure 2G). The expression of ZNF205‐AS1 in EGR4 stably depleted and control SPC‐A1 cells was measured using qPCR. As displayed in Figure 2H, EGR4 knockdown down‐regulated ZNF205‐AS1. Collectively, these results suggested that EGR4 directly bound to the promoter of *ZNF205‐AS1* and activated the transcription of *ZNF205‐AS1*.

### 3.3 EGR4 was increased and positively correlated with ZNF205‐AS1 in NSCLC tissues

To evaluate whether the regulation of ZNF205‐AS1 by EGR4 exist in vivo, the expression of EGR4 in the same 90 pairs of NSCLC tissues and adjacent noncancerous lung tissues used in Figure 1A was measured using qPCR. Consistent with ZNF205‐AS1, EGR4 was also increased in NSCLC tissues compared with adjacent noncancerous lung tissues (Figure 2I). Next, the expression correlation between ZNF205‐AS1 and EGR4 in these 90 NSCLC tissues was calculated. As presented in Figure 2J, the expression of ZNF205‐AS1 was significantly positively correlated with that of EGR4 in NSCLC tissues (*r* = 0.7193, *P* < 0.0001), supporting the positive modulation of ZNF205‐AS1 by EGR4 in vivo.

### 3.4 ZNF205‐AS1 stabilized EGR4 mRNA via RNA‐RNA interaction

**Figure 3:**
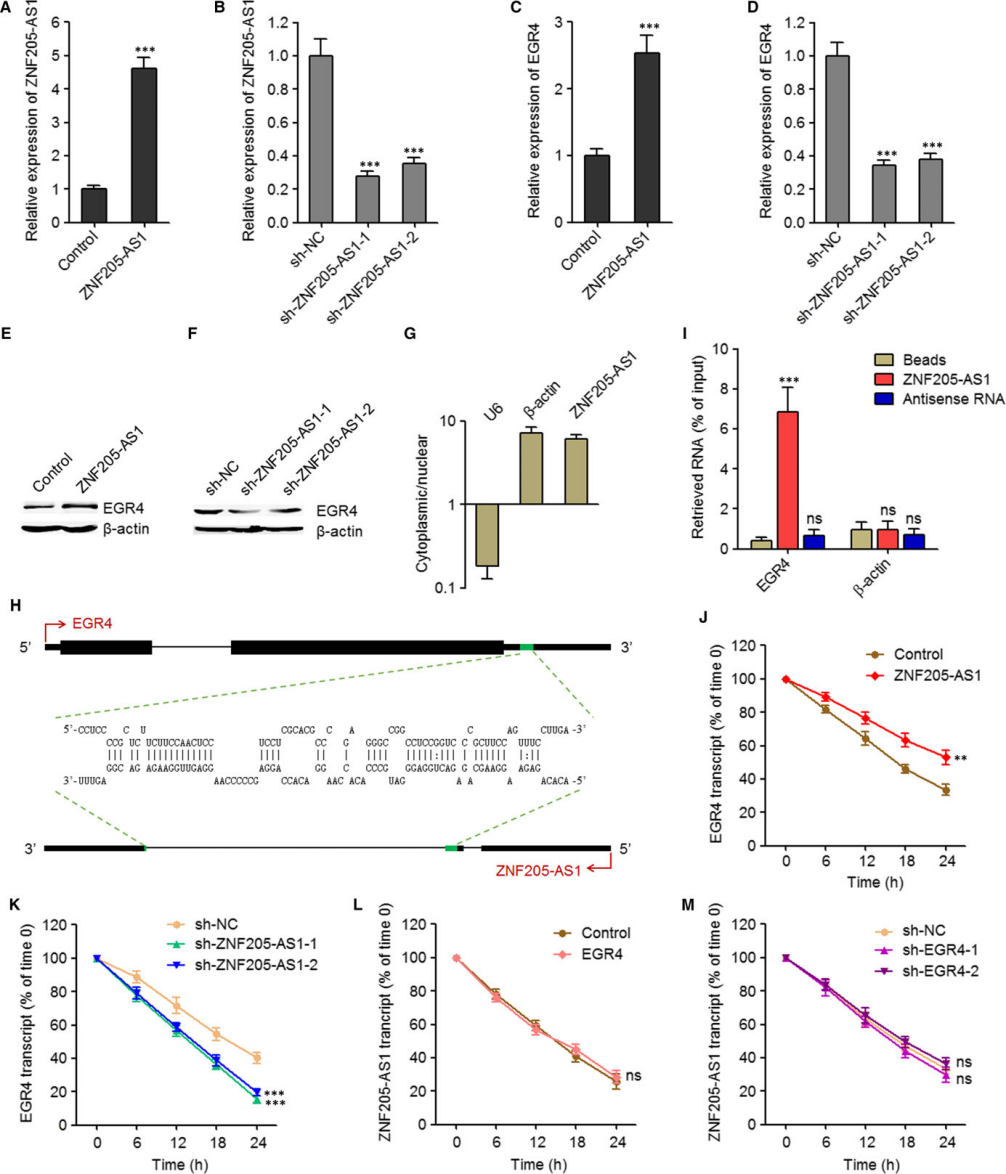
ZNF205‐AS1 stabilized EGR4 mRNA via RNA‐RNA interaction. A, The expression of ZNF205‐AS1 in ZNF205‐AS1 stably overexpressed and control PC‐9 cells was detected by qPCR. B, The expression of ZNF205‐AS1 in ZNF205‐AS1 stably depleted and control SPC‐A1 cells was detected by qPCR. C, The mRNA levels of EGR4 in ZNF205‐AS1 stably overexpressed and control PC‐9 cells were detected by qPCR. D, The mRNA levels of EGR4 in ZNF205‐AS1 stably depleted and control SPC‐A1 cells was detected by qPCR. E, The protein levels of EGR4 in ZNF205‐AS1 stably overexpressed and control PC‐9 cells were detected by western blot. F, The protein levels of EGR4 in ZNF205‐AS1 stably depleted and control SPC‐A1 cells was detected by western blot. G, The subcellular distribution of ZNF205‐AS1 in SPC‐A1 cells was detected by qPCR. U6 and β‐actin were used as nuclear and cytoplasmic control, respectively. H, Schematic diagram of the predicted RNA‐RNA interaction between EGR4 mRNA and ZNF205‐AS1 transcript. I, The RNA‐RNA interaction between EGR4 mRNA and ZNF205‐AS1 transcript was detected by RNA pulldown assay using in vitro transcribed biotin‐labelled ZNF205‐AS1. The retrieved RNA was quantified by qPCR and displayed as percentage of input RNA. J, After transiently transfecting ZNF205‐AS1 overexpression plasmids into PC‐9 cells, the stability of EGR4 mRNA over time was detected by qPCR relative to time 0 after blocking new RNA synthesis with α‐amanitin (50 µM) and normalized to 18S rRNA (transcribed by RNA polymerase I and not influenced by α‐amanitin). K, After transiently transfecting ZNF205‐AS1 specific shRNAs into SPC‐A1 cells, the stability of EGR4 mRNA over time was detected by qPCR relative to time 0 after blocking new RNA synthesis with α‐amanitin (50 µM) and normalized to 18S rRNA. L, After transiently transfecting EGR4 overexpression plasmids into PC‐9 cells, the stability of ZNF205‐AS1 transcript over time was detected by qPCR relative to time 0 after blocking new RNA synthesis with α‐amanitin (50 µM) and normalized to 18S rRNA. M, After transiently transfecting EGR4 specific shRNAs into SPC‐A1 cells, the stability of ZNF205‐AS1 transcript over time was detected by qPCR relative to time 0 after blocking new RNA synthesis with α‐amanitin (50 µM) and normalized to 18S rRNA. Results are displayed as mean ± SD of three independent experiments. **P < 0.01, ***P < 0.001, ns, not significant, by Student's *t* test (A, C, J, L) or one‐way ANOVA followed by Dunnett's multiple comparison test (B, D, I, K, M)

Due to the significant correlation between ZNF205‐AS1 and EGR4 in NSCLC, we next investigated whether ZNF205‐AS1 also modulate EGR4 in NSCLC. We constructed ZNF205‐AS1 stably overexpressed and control PC‐9 cells via transfecting ZNF205‐AS1 overexpression and empty plasmids, respectively. The overexpression efficiency was verified using qPCR (Figure 3A). We also stably depleted ZNF205‐AS1 in SPC‐A1 cells via transfecting two independent ZNF205‐AS1 specific shRNAs. The knockdown efficiency was verified using qPCR (Figure 3B). The mRNA levels of EGR4 in ZNF205‐AS1 stably overexpressed and control PC‐9 cells, and ZNF205‐AS1 stably depleted and control SPC‐A1 cells was measured using qPCR. As displayed in Figure 3C‐D, ectopic expression of ZNF205‐AS1 up‐regulated EGR4 mRNA levels, and while knockdown of ZNF205‐AS1 by two independent shRNAs both down‐regulated EGR4 mRNA levels. Furthermore, the protein levels of EGR4 in ZNF205‐AS1 stably overexpressed and control PC‐9 cells, and ZNF205‐AS1 stably depleted and control SPC‐A1 cells was measured using western blot. As displayed in Figure 3E‐F, ectopic expression of ZNF205‐AS1 up‐regulated EGR4 protein levels, and while knockdown of ZNF205‐AS1 by two independent shRNAs both down‐regulated EGR4 protein levels. Thus, these results suggested that ZNF205‐AS1 up‐regulated EGR4 expression transcriptionally or post‐transcriptionally.

To explore the detailed mechanisms underlying the positive regulation of EGR4 by ZNF205‐AS1, we first detected the subcellular distribution of ZNF205‐AS1 using purification of cytoplasmic and nuclear RNA, followed by qPCR. As displayed in Figure 3G, ZNF205‐AS1 was mainly located in the cytoplasm. Therefore, we focused our attention on the post‐transcriptional regulation of EGR4 by ZNF205‐AS1. Several cytoplasmic lncRNAs have been shown to regulate the stability and/or expression of target mRNAs via RNA‐RNA interaction.^27^, ^28^ Therefore, we searched the interacted RNAs with ZNF205‐AS1 using IntaRNA (http://rna.informatik.uni-freiburg.de/IntaRNA/Input.jsp).^29^ Intriguingly, 3’UTR of EGR4 mRNA was identified as a direct target of ZNF205‐AS1 with the binding sites at 651‐723 nucleotides of ZNF205‐AS1 and at 1914‐1977 nucleotides of EGR4 mRNA (Figure 3H). To detect the RNA‐RNA interaction between ZNF205‐AS1 and EGR4 mRNA, affinity pulldown of endogenous EGR4 mRNA using in vitro transcribed biotin‐labelled ZNF205‐AS1 was performed. As presented in Figure 3I, ZNF205‐AS1, but not ZNF205‐AS1 antisense RNA, specifically interacted with EGR4 mRNA, but not β‐actin mRNA. Next, we investigated whether the RNA‐RNA interaction between ZNF205‐AS1 and EGR4 mRNA regulate the stability of EGR4 mRNA or ZNF205‐AS1 transcript. We transiently overexpressing ZNF205‐AS1 in PC‐9 cells via transfecting ZNF205‐AS1 overexpression plasmids, and transiently inhibiting ZNF205‐AS1 in SPC‐A1 cells via transfecting ZNF205‐AS1 specific shRNAs. Then, the transfected cells were treated with α‐amanitin to stop new RNA synthesis and the loss of EGR4 mRNA over time was measured. As presented in Figure 3J‐K, ectopic expression of ZNF205‐AS1 elongated the half‐life of EGR4 mRNA, and while knockdown of ZNF205‐AS1 by two independent shRNAs both shortened the half‐life of EGR4 mRNA, suggesting that ZNF205‐AS1 increased EGR4 mRNA stability. In addition, we transiently overexpressing EGR4 in PC‐9 cells via transfecting EGR4 overexpression plasmids, and transiently inhibiting EGR4 in SPC‐A1 cells via transfecting EGR4 specific shRNAs. Then, the transfected cells were treated with α‐amanitin to stop new RNA synthesis and the loss of EGR4 mRNA over time was measured. As presented in Figure 3L‐M, neither EGR4 overexpression nor EGR4 knockdown changed the half‐life of ZNF205‐AS1 transcript, suggesting that EGR4 did not regulate ZNF205‐AS1 transcript stability. Collectively, these results suggested that ZNF205‐AS1 directly bound and stabilized EGR4 mRNA.

### 3.5 The autoregulatory loop of ZNF205‐AS1

**Figure 4:**
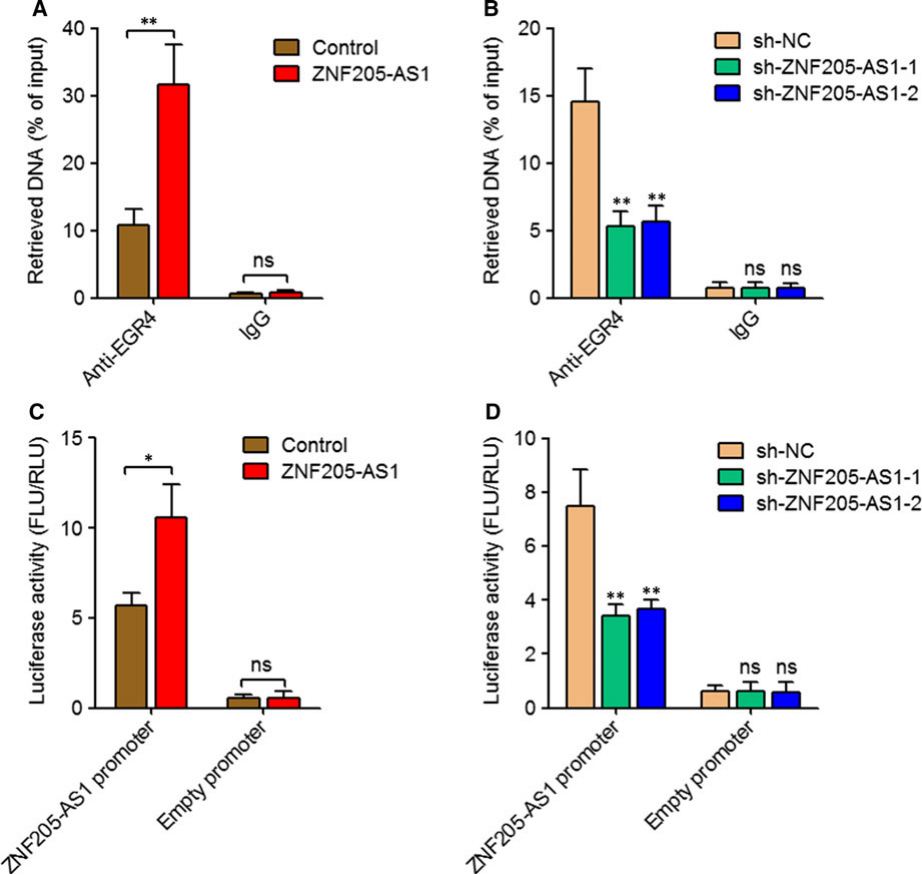
The autoregulatory loop of ZNF205‐AS1. A, After transiently transfecting ZNF205‐AS1 overexpression plasmids into PC‐9 cells, ChIP assay was carried out using EGR4 specific antibody. The retrieved DNA was quantified by qPCR to detect the occupation of EGR4 on the promoter of *ZNF205‐AS1*. Results are displayed as percentage of input DNA. B, After transiently transfecting ZNF205‐AS1 specific shRNAs into SPC‐A1 cells, ChIP assay was carried out using EGR4 specific antibody. The retrieved DNA was quantified by qPCR to detect the occupation of EGR4 on the promoter of *ZNF205‐AS1*. Results are displayed as percentage of input DNA. C, Luciferase reporter assay in PC‐9 cells cotransfected with the *ZNF205‐AS1* promoter reporter construct and ZNF205‐AS1 overexpression plasmid. The ratios of firefly luciferase (FLU) activity to Renilla luciferase (RLU) activity are displayed. D, Luciferase reporter assay in SPC‐A1 cells cotransfected with the *ZNF205‐AS1* promoter reporter construct and ZNF205‐AS1 specific shRNAs. The ratios of firefly luciferase (FLU) activity to Renilla luciferase (RLU) activity are displayed. Results are displayed as mean ± SD of three independent experiments. *P < 0.05, **P < 0.01, ns, not significant, by Student's *t* test (A, C) or one‐way ANOVA followed by Dunnett's multiple comparison test (B, D)

The above results demonstrated that EGR4 activated *ZNF205‐AS1* transcription, and while ZNF205‐AS1 also up‐regulated EGR4, implying a positive feedback loop between ZNF205‐AS1 and EGR4. We next explored whether aberrant elevation of ZNF205‐AS1 in NSCLC feedback regulate its own transcription. After transiently overexpressing ZNF205‐AS1 in PC‐9 cells via transfecting ZNF205‐AS1 overexpression plasmids, ChIP assays were performed, and the results revealed that ectopic expression of ZNF205‐AS1 increased the occupation of EGR4 on *ZNF205‐AS1* promoter (Figure 4A). Conversely, after transiently inhibiting ZNF205‐AS1 in SPC‐A1 cells via transfecting ZNF205‐AS1 specific shRNAs, ChIP assays revealed that ZNF205‐AS1 knockdown decreased the occupation of EGR4 on *ZNF205‐AS1* promoter (Figure 4B). Furthermore, the pGL3 firefly luciferase reporter containing *ZNF205‐AS1* promoter was cotransfected with ZNF205‐AS1 overexpression or empty plasmids into PC‐9 cells. As presented in Figure 4C, ectopic expression of ZNF205‐AS1 increased luciferase activity of the reporter containing *ZNF205‐AS1* promoter. The pGL3 firefly luciferase reporter containing *ZNF205‐AS1* promoter was cotransfected with two independent ZNF205‐AS1 specific shRNAs into SPC‐A1 cells. The results displayed that knockdown of ZNF205‐AS1 by two independent shRNAs both decreased luciferase activity of the reporter containing *ZNF205‐AS1* promoter (Figure 4D). Collectively, these results suggested that ZNF205‐AS1 activated its own promoter activity via promoting the occupation of EGR4 on its own promoter.

### 3.6 ZNF205‐AS1 promoted NSCLC cell growth

**Figure 5:**
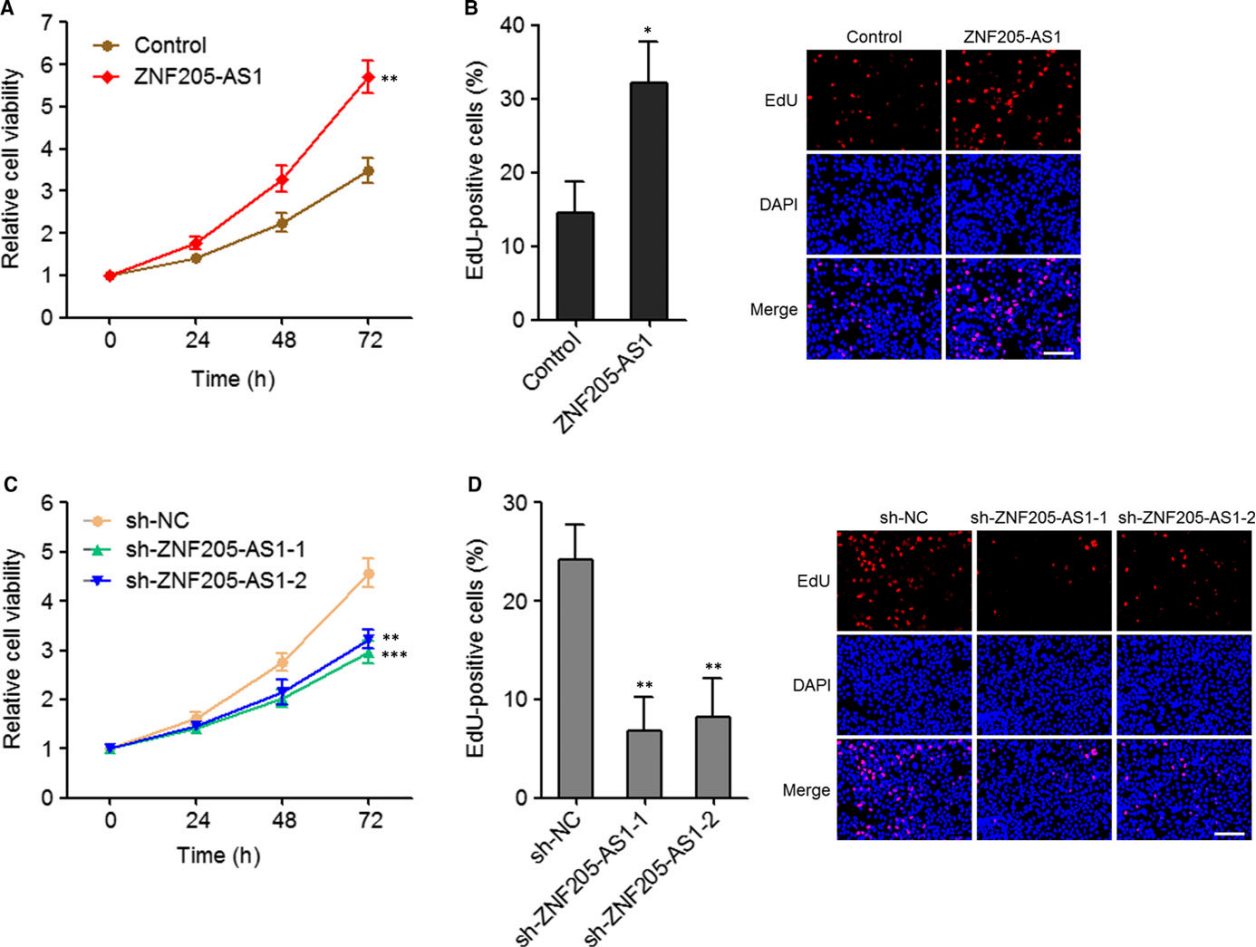
ZNF205‐AS1 promoted NSCLC cell growth. A, Cell viabilities of ZNF205‐AS1 stably overexpressed and control PC‐9 cells were detected using Glo cell viability assay. The relative cell viability to 0 h is presented. B, Cell growth of ZNF205‐AS1 stably overexpressed and control PC‐9 cells were detected using EdU immunofluorescence staining; scale bars = 100 µm. C, Cell viabilities of ZNF205‐AS1 stably depleted and control SPC‐A1 cells were detected using Glo cell viability assay. The relative cell viability to 0 hour is presented. D, Cell growth of ZNF205‐AS1 stably depleted and control SPC‐A1 cells were detected using EdU immunofluorescence staining; scale bars = 100 µm. Results are displayed as mean ± SD of three independent experiments. *P < 0.05, **P < 0.01, ***P < 0.001, by Student's *t* test (A, B) or one‐way ANOVA followed by Dunnett's multiple comparison test (C, D)

Next, we explored the biological roles of ZNF205‐AS1 in NSCLC. To evaluate cell growth potential, Glo cell viability assay and EdU immunofluorescence staining were carried out in ZNF205‐AS1 stably overexpressed and control PC‐9 cells and ZNF205‐AS1 stably depleted and control SPC‐A1 cells. Glo cell viability assay displayed that ectopic expression of ZNF205‐AS1 up‐regulated cell viability of PC‐9 cells (Figure 5A). EdU immunofluorescence staining displayed that ectopic expression of ZNF205‐AS1 increased EdU‐positive and proliferative cells (Figure 5B). Conversely, knockdown of ZNF205‐AS1 by two independent shRNAs both down‐regulated cell viability of SPC‐A1 cells (Figure 5C), and reduced EdU‐positive and proliferative cells (Figure 5D). Collectively, these results demonstrated that ZNF205‐AS1 promoted NSCLC cell growth in vitro.

### 3.7 EGR4 promoted NSCLC cell growth

**Figure 6:**
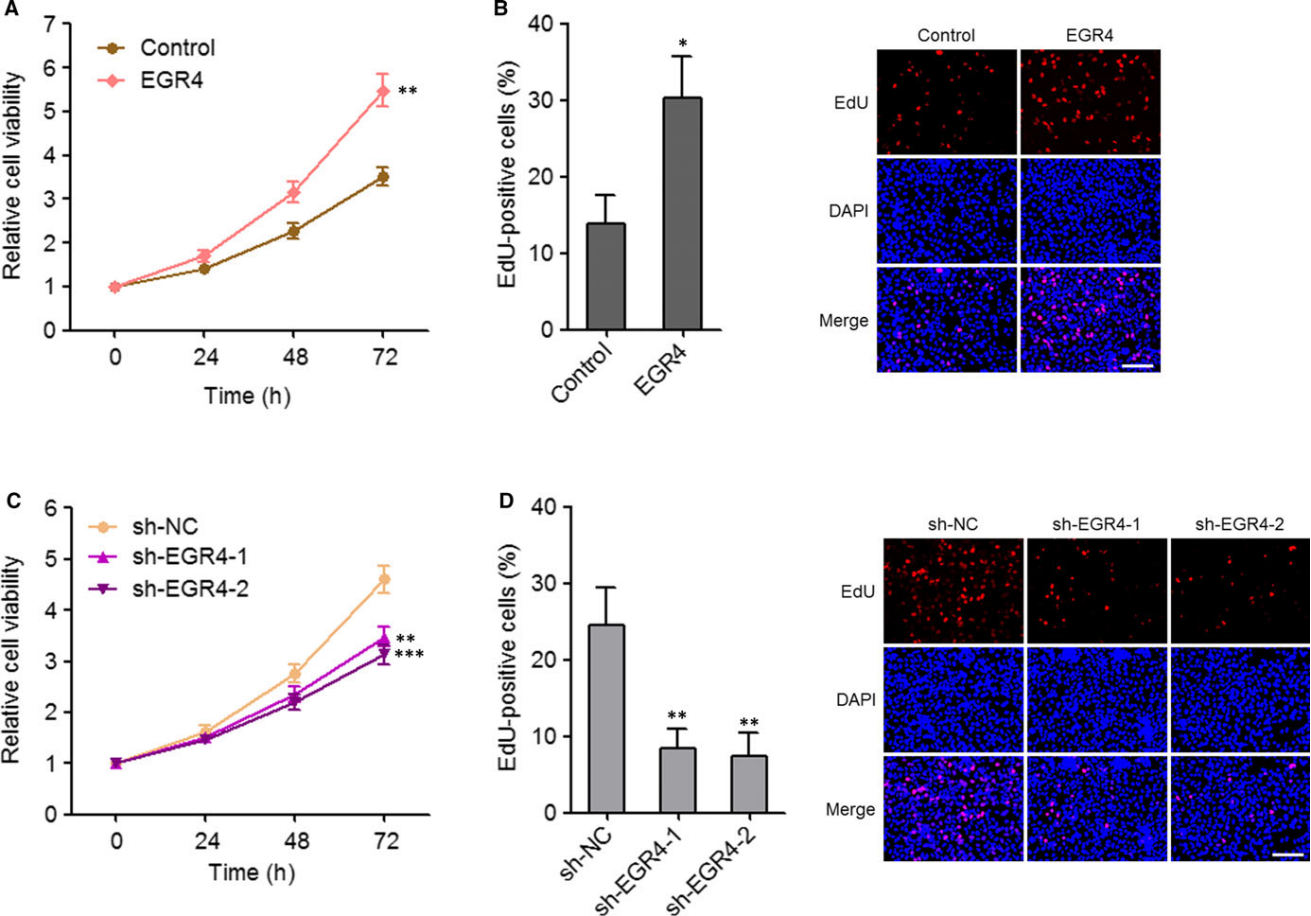
EGR4 promoted NSCLC cell growth. A, Cell viabilities of EGR4 stably overexpressed and control PC‐9 cells were detected using Glo cell viability assay. The relative cell viability to 0 h is presented. B, Cell growth of EGR4 stably overexpressed and control PC‐9 cells were detected using EdU immunofluorescence staining; scale bars = 100 µm. C, Cell viabilities of EGR4 stably depleted and control SPC‐A1 cells were detected using Glo cell viability assay. The relative cell viability to 0 h is presented. D, Cell growth of EGR4 stably depleted and control SPC‐A1 cells were detected using EdU immunofluorescence staining; scale bars = 100 µm. Results are displayed as mean ± SD of three independent experiments. *P < 0.05, **P < 0.01, ***P < 0.001, by Student's *t* test (A, B) or one‐way ANOVA followed by Dunnett's multiple comparison test (C, D)

Due to ZNF205‐AS1 and EGR4 positively regulated each other, we further explored the biological roles of EGR4 in NSCLC. Similarly, Glo cell viability assay and EdU immunofluorescence staining were carried out in EGR4 stably overexpressed and control PC‐9 cells and EGR4 stably depleted and control SPC‐A1 cells. Glo cell viability assay displayed that ectopic expression of EGR4 up‐regulated cell viability of PC‐9 cells (Figure 6A). EdU immunofluorescence staining displayed that ectopic expression of EGR4 increased EdU‐positive and proliferative cells (Figure 6B). Conversely, knockdown of EGR4 by two independent shRNAs both down‐regulated cell viability of SPC‐A1 cells (Figure 6C), and reduced EdU‐positive and proliferative cells (Figure 6D). Collectively, these results demonstrated that consistent with ZNF205‐AS1, EGR4 also promoted NSCLC cell growth in vitro.

### 3.8 Targeting the positive feedback loop between ZNF205‐AS1 and EGR4 inhibited NSCLC tumour growth in vivo

**Figure 7:**
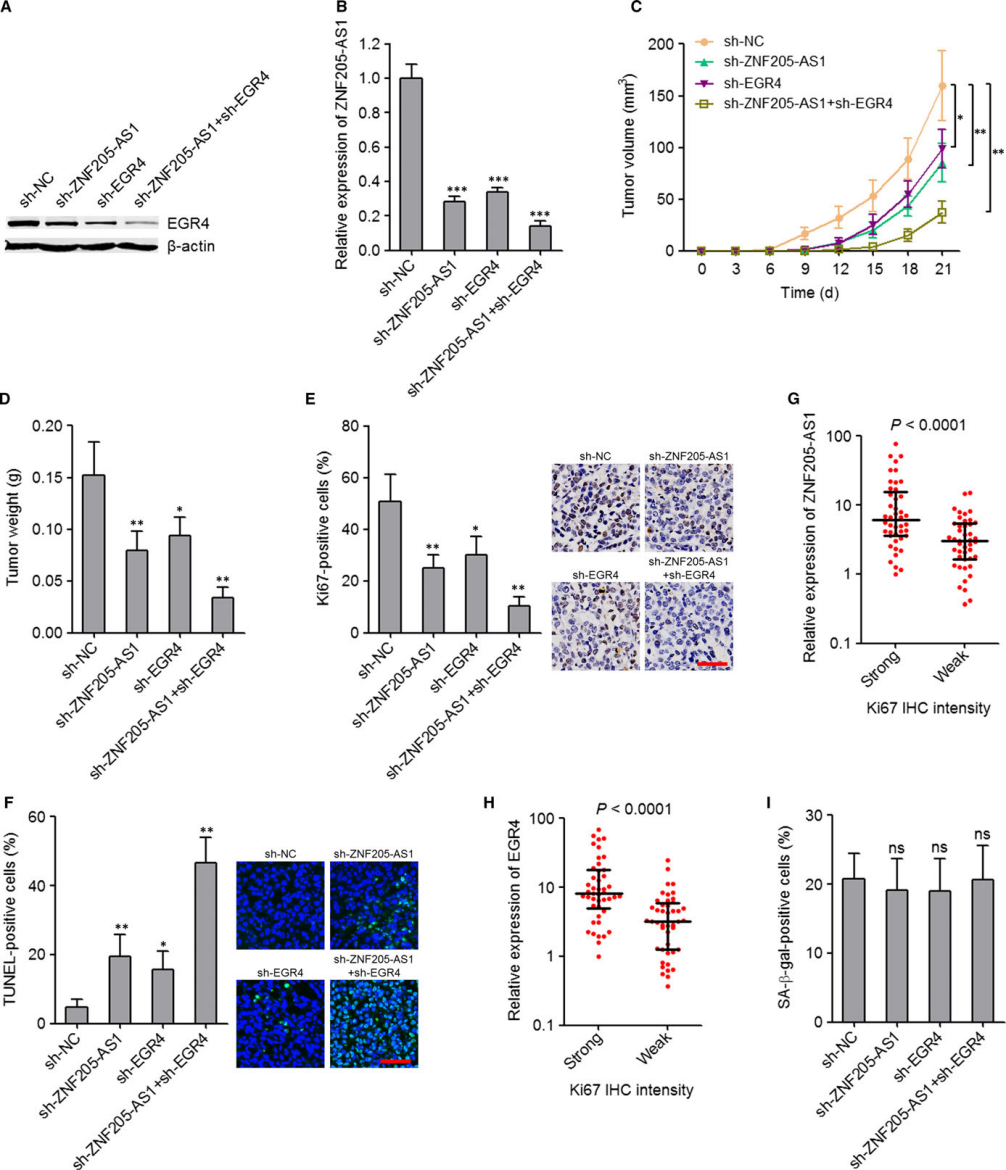
Inhibition of the positive feedback loop between ZNF205‐AS1 and EGR4 significantly repressed NSCLC tumour growth in vivo. A, The expression of EGR4 in ZNF205‐AS1 and EGR4 concurrently depleted and control SPC‐A1 cells was detected by western blot. B, The expression of ZNF205‐AS1 in ZNF205‐AS1 and EGR4 concurrently depleted and control SPC‐A1 cells was detected by qPCR. Results are displayed as mean ± SD of three independent experiments. ***P < 0.001 by one‐way ANOVA followed by Dunnett's multiple comparison test. C, ZNF205‐AS1 and EGR4 concurrently depleted and control SPC‐A1 cells were subcutaneously injected into nude mice. Tumour volumes were detected every three days. D, Subcutaneous tumour weights were detected at the 21th day after injection. E, Ki67 immunohistochemistry staining of tumours derived from ZNF205‐AS1 and EGR4 concurrently depleted or control SPC‐A1 cells; scale bars = 50 μm. F, TUNEL staining of tumours derived from ZNF205‐AS1 and EGR4 concurrently depleted or control SPC‐A1 cells; scale bars = 50 μm. For C‐F, results are displayed as mean ± SD of n = 5 mice in each group. *P < 0.05, **P < 0.01 by Mann‐Whitney test. G,H, ZNF205‐AS1 (G) and EGR4 (H) expression levels in NSCLC tissues with strong or weak Ki67 staining intensity. The median Ki67 staining intensity was used as the cutoff (n = 90 NSCLC tissues). Results are displayed as median with interquartile range. *P* < 0.0001 by Mann‐Whitney test. I, Cellular senescence of ZNF205‐AS1 and EGR4 concurrently depleted and control SPC‐A1 cells was detected by SA‐β‐gal activity. ns, not significant, by one‐way ANOVA followed by Dunnett's multiple comparison test

ZNF205‐AS1 and EGR4 formed a positive feedback loop in NSCLC, and both ZNF205‐AS1 and EGR4 promoted NSCLC cell growth in vitro. We next investigated the significances of targeting the positive feedback loop between ZNF205‐AS1 and EGR4 for NSCLC. We constructed ZNF205‐AS1 and EGR4 concurrently stably depleted SPC‐A1 cells via transfecting ZNF205‐AS1 and EGR4 specific shRNAs. The knockdown efficiency of EGR4 was confirmed using western blot (Figure 7A). The knockdown efficiency of ZNF205‐AS1 was confirmed using qPCR (Figure 7B). ZNF205‐AS1 and EGR4 concurrently depleted and control SPC‐A1 cells were subcutaneously implanted into nude mice. Subcutaneous tumour growth was detected every three days (Figure 7C). Subcutaneous tumours were excised and weighed at the 21th day after injection (Figure 7D). As presented in Figure 7C‐D, ZNF205‐AS1 or EGR4 knockdown both repressed subcutaneous tumour growth. The concurrent knockdown of ZNF205‐AS1 and EGR4 more significantly repressed tumour growth. Proliferation marker Ki67 IHC staining of subcutaneous xenografts further supported the growth repressive roles of ZNF205‐AS1 or EGR4 knockdown, and more significant growth repressive roles of concurrent ZNF205‐AS1 and EGR4 knockdown (Figure 7E). Apoptosis marker TUNEL staining of subcutaneous xenografts displayed that ZNF205‐AS1 or EGR4 knockdown both promoted cell apoptosis of subcutaneous tumours, and concurrent knockdown of ZNF205‐AS1 and EGR4 more significantly promoted cell apoptosis of subcutaneous tumours (Figure 7F). Furthermore, we measured Ki67 expression in the same 90 NSCLC tissues used in Figures 1A and 2I by IHC staining. The expression of ZNF205‐AS1 and EGR4 was both higher in the NSCLC tissues with strong Ki67 staining intensity than that with weak Ki67 staining intensity (Figure 7G‐H). To investigate whether cellular senescence is implicated in the growth‐inhibitory roles of targeting ZNF205‐AS1 and EGR4, cellular senescence of ZNF205‐AS1 and EGR4 concurrently depleted and control SPC‐A1 cells was determined using SA‐β‐gal staining. As presented in Figure 7I, neither ZNF205‐AS1 nor EGR4 knockdown changed senescence of SPC‐A1 cells. Collectively, these results suggested that targeting the positive feedback loop between ZNF205‐AS1 and EGR4 inhibited NSCLC tumour growth in vivo, but did not regulate senescence.

## 4 DISCUSSION

Genome and transcriptome sequencings have found more than 58 000 lncRNAs in human cells, but the number of protein‐coding genes is only about 21 000, implying that the lncRNA landscape may be more complex and various.^30^ Indeed, many aberrantly expressed lncRNAs have been identified in cancers.^31^ Most of lncRNAs are temporal‐spacial specifically and disease specifically expressed.^32^ Although several lncRNAs have been found to play roles in cancers and regarded as cancer‐associated lncRNAs,^33^ the roles of most of these 58 000 lncRNAs in cancers are still unclear. Using siRNAs library against lncRNAs, Seiler et  al performed a functional screen to search potential lncRNAs regulating lung cancer cell viability.^22^ LncRNA ZNF205‐AS1 was identified as a candidate. In this study, we further investigate the expression and biological roles of ZNF205‐AS1 in NSCLC. We found that ZNF205‐AS1 was increased in NSCLC tissues and cell lines compared with adjacent noncancerous lung tissues and normal bronchial epithelial cell line, respectively. Furthermore, increased expression of ZNF205‐AS1 was positively associated with poor pathological differentiation, great tumour diameter, lymph nodes metastasis, advanced TNM stage, and poor overall survival of NSCLC patients. These data implied that ZNF205‐AS1 may be implicated in the progression of NSCLC. Gain‐of‐function and loss‐of‐function assays revealed that ZNF205‐AS1 promoted NSCLC cell growth in vitro, and NSCLC tumour growth in vivo. These results demonstrated that ZNF205‐AS1 functioned as an oncogene in NSCLC and ZNF205‐AS1 may also be regarded as a cancer‐associated lncRNA in NSCLC.

Although many aberrantly expressed lncRNAs have been identified in cancers, the reasons contributing to the dysregulation of lncRNAs are relatively less studied. In this study, using computational screen, we identified EGR4 binding sites on the promoter of *ZNF205‐AS1*. EGR4 is a transcription factor, which belongs to the early growth response (EGR) family of immediate early genes.^34^ Previous study mainly focused on the roles of EGR4 in male infertility and neural development.^35^, ^36^ Matsuo et al reported that EGR4 promoted small cell lung cancer cell proliferation,^25^ but the expression and roles of EGR4 in NSCLC are still unknown. In this study, we first verified the binding of EGR4 to *ZNF205‐AS1* promoter using ChIP. Dual luciferase reporter assays revealed that EGR4 up‐regulated promoter activity of *ZNF205‐AS1*. qPCR verified that EGR4 activated the transcription of *ZNF205‐AS1*. Thus, EGR4 bound to *ZNF205‐AS1* promoter and activated *ZNF205‐AS1* transcription. Consistent with ZNF205‐AS1, EGR4 was also increased in NSCLC tissues, and the expression of EGR4 was significantly positively correlated with that of ZNF205‐AS1 in NSCLC tissues, supporting the positive regulation of ZNF205‐AS1 by EGR4. Functional assays revealed that consistent with ZNF205‐AS1, EGR4 also promoted NSCLC cell growth in vitro, and NSCLC tumour growth in vivo.

Except the positive regulation of ZNF205‐AS1 by EGR4, we further found that ZNF205‐AS1 also up‐regulated the expression of EGR4. Using bioinformatics prediction, we identified a 72 bp interaction region between ZNF205‐AS1 transcript and EGR4 mRNA 3’UTR. RNA pulldown assays verified the binding between ZNF205‐AS1 transcript and EGR4 mRNA 3’UTR. We further verified that ZNF205‐AS1 increased EGR4 mRNA stability, and therefore up‐regulated EGR4 expression. microRNAs (miRNAs) are well‐known to bind the 3’UTR of target mRNAs and induce the degradation and/or translation inhibition of target mRNAs.^37^, ^38^, ^39^, ^40^ The interaction between ZNF205‐AS1 transcript and EGR4 mRNA 3’UTR may protect EGR4 mRNA from miRNAs‐induced degradation, which need further investigation.

EGR4 transcriptionally activated ZNF205‐AS1, and while ZNF205‐AS1 increased EGR4 mRNA stability and up‐regulated EGR4 expression. Thus, EGR4 and ZNF205‐AS1 positively regulated each other and formed a positive feedback loop. Indeed, our data also found that ZNF205‐AS1 up‐regulated the promoter activity of *ZNF205‐AS1* via promoting the binding of EGR4 to *ZNF205‐AS1* promoter, supporting the autoregulatory loop of ZNF205‐AS1. The feedback loops have been reported in many cancers, such as the positive feedback loop between miR‐181b and STAT3 in colon cancer,^41^ the negative feedback loop between miR‐200a and HDAC4 in hepatocellular carcinoma.^42^ Feedback loops could amplify the effects of interaction molecules in caners and more significantly promote the aberrant expression of these molecules.^43^ In this study, we further found that targeting the feedback loop between EGR4 and ZNF205‐AS1 via concurrently depleting EGR4 and ZNF205‐AS1 significantly repressed NSCLC tumour growth in vivo. Although concurrent depletion of EGR4 and ZNF205‐AS1 for clinical application is difficult until now, but the combination of EGR4 inhibitor and ZNF205‐AS1 siRNA therapeutics in the future may be promising therapeutic strategy for NSCLC.

In conclusion, this study demonstrated that lncRNA ZNF205‐AS1 formed a positive feedback loop with EGR4, which contributed to the up‐regulations and the oncogenic roles of ZNF205‐AS1 and EGR4 in NSCLC. Our data suggested that the positive feedback loop between ZNF205‐AS1 and EGR4 may be promising therapeutic target for NSCLC.

## CONFLICT OF INTEREST

The authors declare that they have no conflict of interest.
